# Secondary Prevention of AFAIS: Deploying Traditional Regression, Machine Learning, and Deep Learning Models to Validate and Update CHA2DS2-VASc for 90-Day Recurrence

**DOI:** 10.3390/jcm14207327

**Published:** 2025-10-16

**Authors:** Jenny Simon, Łukasz Kraiński, Michał Karliński, Maciej Niewada, on behalf of the VISTA-Acute Collaboration

**Affiliations:** 1 Department of Experimental and Clinical Pharmacology, Medical University of Warsaw, 02-091 Warsaw, Poland; maciej.niewada@wum.edu.pl; 2 School of Mathematics and Statistics, The Open University, Milton Keynes MK7 6AA, UK; 3 SGH Warsaw School of Economics, 02-554 Warsaw, Poland; lkrain@sgh.waw.pl; 4 2nd Department of Neurology, Institute of Psychiatry and Neurology, 02-957 Warsaw, Poland; mkarlinski@ipin.edu.pl

**Keywords:** atrial fibrillation, stroke risk prediction, secondary prevention, oral anticoagulants, clinical prediction rule

## Abstract

**Backgrounds/Objectives:** Atrial fibrillation (AF) confers a fivefold greater risk of acute ischaemic stroke (AIS) relative to normal sinus rhythm. Among patients with AF-related AIS (AFAIS), recurrence is common: AFAIS rate is sixfold higher in secondary versus primary prevention patients. Guidelines recommend oral anticoagulation for primary and secondary prevention on the basis of CHA_2_DS_2_-VASc. However, guideline adherence is poor for secondary prevention. This is, in part, because the predictive value of CHA_2_DS_2_-VASc has not been ascertained with respect to recurrence: patients with and without previous stroke were not routinely differentiated in validation studies. We put forth a protocol to (1) validate, and (2) update CHA_2_DS_2_-VASc for secondary prevention, aiming to deliver a CPR that better captures 90-day recurrence risk for a given AFAIS patient. Overwhelmingly poor quality of reporting has been deplored among published clinical prediction rules (CPRs). Combined with the fact that machine learning (ML) and deep learning (DL) methods are rife with challenges, registered protocols are needed to make the CPR literature more validation-oriented, transparent, and systematic. This protocol aims to lead by example for prior planning of primary and secondary analyses to obtain incremental predictive value for existing CPRs. **Methods:** The Virtual International Stroke Trials Archive (VISTA), which has compiled data from 38 randomised controlled trials (RCTs) in AIS, was screened for patients that (1) had an AF diagnosis, and (2) were treated with vitamin K antagonists (VKAs) or without any antithrombotic medication. This yielded 2763 AFAIS patients. Patients without an AF diagnosis were also retained under the condition that they were treated with VKAs or without any antithrombotic medication, which yielded 7809 non-AF AIS patients. We will validate CHA_2_DS_2_-VASc for 90-day recurrence and secondary outcomes (7-day recurrence, 7- and 90-day haemorrhagic transformation, 90-day decline in functional status, and 90-day all-cause mortality) by examining discrimination, calibration, and clinical utility. To update CHA_2_DS_2_-VASc, logistic regression (LR), extreme gradient boosting (XGBoost), and multilayer perceptron (MLP) models will be trained using nested cross-validation. The MLP model will employ transfer learning to leverage information from the non-AF AIS patient cohort. **Results:** Models will be assessed on a hold-out test set (25%) using area under the receiver operating characteristic curve (AUC), calibration curves, and F1 score. Shapley additive explanations (SHAP) will be used to interpret the models and construct the updated CPRs. **Conclusions:** The CPRs will be compared by means of discrimination, calibration, and clinical utility. In so doing, the CPRs will be evaluated against each other, CHA_2_DS_2_-VASc, and default strategies, with test tradeoff analysis performed to balance ease-of-use with clinical utility.

## 1. Introduction

Atrial fibrillation (AF) is the most common cardiac arrhythmia [1] and confers a fivefold greater risk of acute ischaemic stroke (AIS) relative to normal sinus rhythm [2]. AF-related AIS (AFAIS) is reported to account for 13–26% of global AIS burden [3], and its association with greater infarction size, severity, disability, mortality, and cost compared to AIS of non-thromboembolic pathogenesis is well-documented [4,5,6,7,8]. Fortunately, AFAIS is known to be highly preventable with oral anticoagulants (OACs) [9]. Current guidelines recommend OACs for primary and secondary prevention on the basis of CHA_2_DS_2_-VASc [10,11], a prognostic clinical prediction rule (CPR) that estimates annual risk of AFAIS. However, it has been reported that only 32% of AFAIS survivors are prescribed OACs for secondary prevention in a guideline-adherent manner [12,13]. In fact, several studies have shown that patients with higher CHA_2_DS_2_-VASc scores were paradoxically less likely to receive antithrombotic therapy [1,14,15]. While this ‘anticoagulation paradox’ is partly attributable to the fact that a high CHA_2_DS_2_-VASc score implies high bleeding risk [16], there is also concern that OACs are being omitted for non-therapeutic reasons despite RCT evidence substantiating that recurrence rates are highly modifiable with OACs [17]. This is alarming given that 1-year recurrence rates have been reported to be as high as 30% [18,19]. We contend that the absence of research ascertaining the predictive performance and clinical utility of CHA_2_DS_2_-VASc with regard to recurrence is partly to blame. Indeed, it is a major limitation that patients with and without stroke history were not routinely differentiated in studies validating CHA_2_DS_2_-VASc [20]. Rather, validation studies employed numerical scores to divide subjects into risk strata irrespective of whether a score of 4 belonged to a 75-year-old female patient with diabetes or a 60-year-old male with history of hypertension, stroke, and myocardial infarction, for example. Therefore, data on the performance of CHA_2_DS_2_-VASc among cohorts with previous AIS is scarce, and existing studies have reported poor discrimination, with C-statistics generally below 0.6 [21]. This prompted us to design an external validation study for CHA_2_DS_2_-VASc in AF patients scoring ≥ 2 points after having suffered an AIS.

To date, one study has analysed stratum-specific relative risk of mortality associated with unique combinations of C, H, A, D, and S in the since-superseded CHADS_2_ score and found S_3_—assigning 3 points to stroke history—to be a better fit to available mortality data than S_2_ [22]. A more recent study expanded on CHA_2_DS_2_-VASc with additional comorbid features to yield a multi-morbid index that reported that the weight for stroke history should be increased to 19 (out of a total of 44) points [23]. In fact, one well-known ‘blind spot’ of CHA_2_DS_2_-VASc is that a patient with a history of stroke and no other risk factor is assigned a score of 2 corresponding to an annual stroke risk of 2.2–4.5% [24,25], which underestimates recurrence rates. These findings suggest that secondary prevention may merit a bespoke CPR. To this end, we also set out to update CHA_2_DS_2_-VASc to yield a CPR that is better suited to the acute context of AFAIS. We deploy traditional regression as well as machine learning (ML) and deep learning (DL) tools to more aptly capture the relative importances of the constituent features of CHA_2_DS_2_-VASc in the context of early secondary prevention, when the risk of recurrence is highest.

Timing of OAC initiation is a critical aspect of secondary prevention. The early post-stroke period is characterised by a transient state of heightened vulnerability to both thromboembolism and haemorrhage, necessitating individualised assessment of risk of recurrence versus haemorrhagic transformation (HT). At present, the timing of OAC administration is founded on expert opinion and observational data (level C evidence) [26]. Evocative of the absence of consensus was a survey conducted among UK specialists which reported that 95% of respondents were uncertain about optimal timing for novel OAC (NOAC) (re)introduction [27]. This is a consequence of the historical exclusion of AIS patients from major phase III RCTs in the context of stroke prevention in AF (SPAF). Patients were excluded for at least 7 days after AIS in the ARISTOTLE trial (apixaban) [28], 14 days after minor AIS and 90 days after major AIS in the ROCKET-AF trial (rivaroxaban) [29], and 14 days after minor AIS and 180 days after major AIS in the RE-LY trial (dabigatran) [30]. To address this critical gap, four RCTs—ELAN [31], TIMING [32], START [33], and OPTIMAS [34], collectively enrolling over 9000 participants [35]—investigated the timing of NOAC initiation in AFAIS patients. Of these, two trials—ELAN and TIMING—have been completed. The ELAN trial evaluated early NOAC initiation within 2 days for minor strokes and 6 days for moderate strokes, while the TIMING trial assessed initiation within 4 days for minor strokes and 7–14 days for major strokes. The timeframes ELAN and TIMING set for moderate strokes means that the evidence still does not strongly support very early NOAC initiation (e.g., within 1–2 days) for many patients. The ongoing START and OPTIMAS trials adopted flexible approaches also tailored to stroke severity, generally considering both early and delayed strategies within the first 14 days post-stroke. Against this backdrop, the analyses planned herein will be a timely contribution to the body of literature that aims to better understand early AFAIS recurrence patterns and support the selection of patients who are most likely to benefit from very early OAC.

Deploying well-validated CPRs is one way of implementing personalised evidence-based medicine [36]. CPRs are abundant in the biomedical literature, and their publication has proliferated in recent years [37,38]. The publication of at least 731 diagnostic and prognostic prediction model studies on COVID-19 during the first 12 months of the pandemic is a case in point [39]. Although a great many stroke-risk CPRs have been proposed, CHA_2_DS_2_-VASc is well-validated and commonly used worldwide [40,41], and its use has been recommended in clinical guidelines for over a decade [42]. This is the exception rather than the rule among CPRs, as there is a habit of developing more CPRs for the same purpose in lieu of validating existing ones [43]. Newly developed CPRs are thus often based on smaller samples, and information captured within previous cohorts is neglected, running contrary to the principle that inferences should be founded on the cumulative data of as many patients as possible. As a result, there is remarkably little data to suggest a positive impact on the process or outcome of clinical care despite mushrooming CPRs [37].

Clearly, the ability to critically evaluate new CPRs is key, and is predicated on complete and transparent reporting [44]. Yet numerous studies evaluating CPR publications have found them to be poorly conducted and incompletely reported, with pervasive deficiencies in statistical methods and high risk of bias (ROB) [45,46,47,48]. In particular, small datasets, inappropriate imputation algorithms, and inadequate internal validation techniques were common, and major methodological details were frequently missing [49,50,51,52,53,54,55,56,57,58,59,60,61,62,63,64]. The 2015 TRIPOD guideline was born out of necessity for full and clear reporting of CPRs [65]. Its artificial intelligence (AI) extension, TRIPOD+AI, was introduced in 2024 to offer specialised guidance for the unique challenges posed by ML and DL, including overfitting, interpretability, and the risk of embedding biases [44]. In addition, the development of detailed study protocols at the outset of CPR research is increasingly being recognised as crucial to enhancing transparency and peer scrutiny, facilitating reproducibility and cooperation, and mitigating ROB and creative data dredging [66,67,68,69]. TRIPOD+AI features a new section dedicated to open science principles, including item 18c which asks that researchers indicate where the study protocol can be accessed or state that a protocol was not prepared [44]. The 2024 announcement of the TRIPOD-P guideline, which will focus on the preparation of protocols, marks yet another step forward [70]. In the wait for TRIPOD-P, we hope that this protocol will lead by example for the prior planning of primary and secondary analyses for CPR studies.

Overall, our goal is to develop a CPR with superior discriminative performance, calibration, and clear clinical utility for the early secondary prevention of AFAIS. Among the cardiovascular contributors to stroke investigated in the seminal Framingham Heart Study, AF was unique in that its effect on AIS risk did not abate with advancing age [71]. In fact, AF was the sole cardiovascular condition to exert an independent effect on stroke incidence among patients aged 80–89 years. With demographic ageing, prevalence of AF is projected to increase 2.5-fold over the next 40 years [72] and has already been reported to be on a significant incline among acute hospital admissions [73]. Taking these factors in conjunction, a substantial rise in the societal burden of AFAIS may materialise in coming decades [74,75], underscoring the need for improved secondary prevention. As a matter of fact, the dynamic nature of patient demographics suggests that every CPR is subject to an expiration date [76,77], and we argue the time is ripe to challenge the status quo of CHA_2_DS_2_-VASc. Of course, numerous external validation studies and impact studies will be necessary to determine whether the CPR we develop increases physicians’ guideline-adherence or, on the contrary, obfuscates OAC prescription. Additionally, it will be interesting to explore whether the CPR proves useful for other purposes—guiding neurologist referrals, informing recruitment in RCTs, controlling for confounding variables in observational research (e.g., via propensity scores), and educating patients—that indirectly improve secondary prevention [78]. Parenthetically, we hope researchers forgo the habit of continually developing new CPRs before rigorously validating and updating existing ones and adopt the habit of abiding by open science practices such that the literature may become more validation-oriented, transparent, and systematic.

## 2. Methodology

### 2.1. TRIPOD+AI Adherence

Items of TRIPOD+AI that could be met within the confines of a protocol have been addressed. The checklist has been provided in the Supplementary Materials file S2.

### 2.2. Data Source

The Virtual International Stroke Trials Archive (VISTA) is an international, prospective data repository whose Acute subsection (VISTA-Acute) has collated data from 38 RCTs in AIS to date, the methods of which have previously been described [79]. Study timelines including start and end of accrual were unique to each contributing trial and, where available, have been provided in the appending materials. Study setting and geographic location of centres also differed for each trial and have been provided. These data were presented in aggregate because the number of centres implicated was not disclosed. Eligibility criteria for participants have been compiled across the contributing trials and made available in the appending materials, too. The high quality of the VISTA dataset provides an excellent opportunity for analysis of prognostic factors and has already allowed several stroke-risk prediction models to be established and validated [80].

### 2.3. Study Population

The filters applied in screening the repository were that the patient (1) had an AF diagnosis, and (2) was treated with vitamin K antagonists (VKAs) or without any antithrombotic medication. Criterion (2) was informed by the fact that contributing trials predated the introduction of novel oral anticoagulants (NOACs).

This yielded 2763 patients with documented age; sex; pre-stroke modified Rankin Scale (mRS) score; 90-day mRS; baseline National Institutes of Health Stroke Scale (NIHSS) score; 30- and 90-day NIHSS; medical history including congestive heart failure (CHF), hypertension, hyperlipidaemia, diabetes mellitus (DM), myocardial infarction (MI), coronary artery disease (CAD), transient ischaemic attack (TIA), prior AIS, and tobacco use; intravenous thrombolysis with recombinant tissue plasminogen activator (IVT); and onset-to-thrombolysis time (OTT). Medication administration records and adverse event records were also extracted: the former were screened for start and end days of VKA administration relative to AIS and the latter were screened for 7- and 90-day recurrence; 7- and 90-day haemorrhagic transformation (HT); and 90-day mortality.

Patients without an AF diagnosis were also retained under the condition that they were treated with vitamin K antagonists (VKAs) or without any antithrombotic medication. This yielded 7809 patients, whose records documented the same information as above.

The AF cohort will be used for primary and secondary analyses, while the non-AF cohort will serve exclusively for secondary analyses.

### 2.4. Predictor Variables

The predictor variable in the validation analyses is CHA_2_DS_2_-VASc score, computed on the basis of its constituent features. It is computed as follows: 1 point for each of CHF, hypertension, DM, vascular disease (MI, CAD, or peripheral artery disease), age 65–74 years, and sex category (female), and 2 points for each of age ≥ 75 years and antecedents of AIS or TIA [24]. All features were measured at baseline, and, with the exception of age, all were binary encoded. As one of the more subjective items considered by CHA_2_DS_2_-VASc, peripheral artery disease was seldom recorded by contributing trials. Hence, MI and CAD will underlie the vascular disease feature.

The predictor variables in the updating analyses are the constituent features of CHA_2_DS_2_-VASc—omitting S_2_, which provides no discriminatory information in a cohort where all patients inherently have the same value (i.e., 2)—and previous stroke. Crucially, since secondary prevention is, by definition, aimed at patients having sustained an AIS, previous stroke is defined herein as any episode of AIS *prior* to the one that prompted recruitment. Besides preserving the familiar 7-feature structure of CHA_2_DS_2_-VASc, incorporating this variable in the updated CPR is very apropos in the modern-day context of effective treatment and demographic ageing which signify that many patients survive one or more strokes.

### 2.5. Confounding Variables

The measured confounding variables are antithrombotic treatment (VKAs or none), start and end days of VKA administration, IVT, and OTT. Antithrombotic medication, IVT, and the timings of their administration constitute all information available about treatments received by participants.

Actions taken to blind the assessment of predictors or confounding variables in contributing trials, if any, were not recorded by VISTA. Qualifications and demographic characteristics of predictor assessors were not available.

### 2.6. Outcome Variables

The primary outcome variables are 90-day recurrence, defined as any episode of AIS within 90 days of the event that prompted recruitment. Of our 2763 AFAIS patients (and 7809 non-AF AIS patients), some will have no prior history of stroke while others will have suffered antecedents. Thus, our primary outcome variable includes second as well as third or *n*th strokes.

The secondary outcome variables are 7-day recurrence; 7- and 90-day HT; 90-day decline in functional status; and 90-day all-cause mortality. The 7-day recurrence is defined analogously to the primary outcome variable: any episode of AIS within 7 days of the event that prompted recruitment. The 7- and 90-day HT are defined as any episode of intracranial haemorrhage (ICH) within 7 and 90 days of the event that prompted recruitment, respectively.

Decline in functional status is defined as 90-day mRS score ≥ 2 for patients that were not disabled before stroke (pre-stroke mRS 0–1) and failure to return to pre-stroke mRS score at 90 days for patients that were disabled before stroke (pre-stroke mRS ≥ 2). In other words, progression on the scale qualifies as decline in functional status, with the exception of progression from mRS 0 to 1, seeing as even minor residual deficit post-stroke will, by definition, increase an mRS 0 patient to mRS 1.

To account for the fact that death within 90 days may preclude observation of 7- and 90-day recurrence, we will conduct a sensitivity analysis using a Cox proportional hazards model with mortality treated as a censoring event.

All contributing trials were double-blind RCTs, though no further details on blinded outcome assessment were accessible. Qualifications and demographic characteristics of outcome assessors were not available.

### 2.7. Sample Size and Power Analysis

Validating a CPR demands a moderate sample size relative to updating a CPR, which demands a large sample size [78]. Nonetheless, updating a CPR—meaning the predictors are known but not the functional form or model parameters—is still less data-demanding than development from scratch, where even the predictors are unknown. We employed the methods proposed by van Smeden et al. [81,82] and Riley et al. [83] to calculate the required sample sizes for our regression analyses. These methods were formulated to guide power analyses for studies intending to develop models from scratch. They have been encapsulated in a four-step procedure that represents a significant advancement over the historically inconsistent and poorly substantiated rules of thumb regarding the desirable number of events per variable (EPV) [84].

Each of the four steps in this procedure yielded a sample size estimate, with the largest selected as the required sample size. This approach ensured that our sample size was sufficient to accomplish the following goals: (1) estimate the overall 90-day recurrence prevalence with high precision, (2) minimise the mean absolute prediction error, (3) require minimal regularisation, and (4) limit optimism as measured by Nagelkerke’s R^2^. Steps 1, 3, and 4 were implemented using the psampsize package developed by the authors for R [84], while step 2 used the tool available at https://mvansmeden.shinyapps.io/BeyondEPV/ (accessed on 4 January 2024). The calculations—which are based on the estimated prevalence of 90-day recurrence in the target population, the number of predictor variables in our CPR, its anticipated discriminative performance, and the acceptable mean absolute prediction error, among other parameters—have been made explicit in the appending materials. We utilised 90-day recurrence rates within the 0.05–0.25 range, conservatively adopting the rate that produced the largest sample sizes.

The sample sizes derived from steps 1, 2, 3, and 4 were 288, 344, 680, and 459 patients, respectively. We therefore deemed a minimum total sample size of 680 patients necessary for CPR development. Given that our sample size is 2763 patients and the fact that we will perform validation and updating (and not development from scratch), we contend that our regression analyses are adequately powered. Of note, 75% of our dataset (*n* = 2072 patients) will be used for regression model training and the remaining 25% (*n* = 691 patients) will be reserved for testing. The fact our test set alone exceeds the required 680 patients further supports our sample size.

ML and DL techniques are inherently more data-intensive than regression [85]. This is because the number of predictors they consider far exceeds that of regression, even when the same set of predictors is considered, because they examine multiple interaction terms [84]. Unfortunately, guidance for a priori sample size calculations is currently lacking for most ML/DL techniques [86]. However, if closely related data are readily available, it has been suggested that inspecting model learning curves in the related dataset can be valuable to estimate the required sample size for the main dataset [87,88]. We will therefore explore the observed power of our non-regression models by means of learning curves on our non-AF dataset, as well as via a key information theoretic metric: Kullback–Leibler (KL) divergence.

Firstly, we will create multiple subsets from the non-AF dataset (ranging from 500 to 5000 patients, in 500-patient increments) and train our ML models on these subsets. Performance will be evaluated using a fixed test set (25% of the non-AF dataset, *n* = 1952). Learning curves will be plotted to depict performance against training subset size, which will help visualise the point at which performance stabilises, indicating diminishing returns with additional data. If this stabilisation point aligns with the size of the AF dataset training set (*n* = 2072), we will be reassured that our sample size is likely adequate. The similarity between the non-AF and AF datasets, which will have at this stage been assessed via descriptive statistics, will inform the reliability of this inference. Learning curves specific to the AF dataset will be generated to further assess the legitimacy of this inference. This will employ the same process, training ML models on progressively larger subsets of the AF dataset (ranging from 500 to 2000 patients, in 500-patient increments) before plotting performance against training subset size. Importantly, DL models will use an enhanced sample size via transfer learning, leveraging 7809 non-AF patients during pretraining. This strategy is beneficial because it addresses the power law that has been observed for large neural networks, whereby performance improves according to *y* = *x*^a^, with *a* < 0 [89], implying that as the number of training samples (*x*) increases, the generalisation error (*y*) decreases at a slowing rate.

KL divergence will be used to assess the generalisation capability of both ML and DL models by comparing the predictive distributions between training and test subsets of the AF dataset. A small KL divergence will suggest that the training and test sets are similarly representative of the underlying distribution, corroborating that the sample size is adequate. The following rule of thumb will guide our judgement: if KL divergence < 0.01, the two distributions will be deemed almost indistinguishable; values between 0.01 and 0.1 will imply minor differences, and >0.1 will indicate more substantial differences [90].

Post hoc analyses of (observed) power are admittedly substandard. However, the insights gained from these methods will offer valuable insight into our dataset’s sufficiency given the absence of specific guidance at the time of writing [86].

### 2.8. Handling Class Imbalance

To address anticipated class imbalance (recurrence rates within the 0.05–0.25 range), class-weight adjustments will be applied in LR, XGBoost, and MLP models. Decision thresholds will be optimised within validation folds using Youden’s J and F1 score. Predicted probabilities will be calibrated using Platt scaling or isotonic regression, with the choice depending on sample size and distributional characteristics (Platt for smaller samples, isotonic when sufficient data allows). Thresholds used in decision curve analysis will be derived from these probabilities calibrated on validation data to ensure clinically meaningful comparisons.

### 2.9. Ethical Comment and Data Availability

Ethical review and approval were waived for this study due to the anonymised nature of the dataset. Restrictions apply to the availability of the data, which we will access under licence for the sole purpose of this research. This protocol underwent review and received approval from the VISTA-Acute Steering Committee. To access the data, interested parties must likewise submit a proposal to the VISTA-Acute Steering Committee. The dataset was previously inaccessible to all authors.

### 2.10. Code Sharing

The code developed for the study will be made available by the corresponding author upon request, though any code segments that could reveal sensitive aspects of the dataset, such as data cleaning code, will not be shared to comply with VISTA’s data security statement. All analyses will be performed in Python, version 3.9.12, primarily using the scikit-learn 1.7.1 and PyTorch libraries 2.7.0.

### 2.11. VISTA vs. the CHA_2_DS_2_-VASc Development Dataset

The CHA_2_DS_2_-VASc development dataset was extracted from the Euro Heart Survey (EHS) on AF, a multi-centre prospective observational study whose methods have previously been described [91]. Patients were enrolled in EHS if they were 18 years or older and had an electrocardiogram or Holter recording showing AF during the qualifying admission/consultation or in the preceding 12 months. The development study [92] screened EHS for patients with (1) absence of mitral stenosis and previous heart valve surgery, and (2) use of neither VKAs nor heparin at discharge. This yielded 1577 patients documenting all of the variables we have access to, with the addition of PAD. Outcome variables were thromboembolism (defined as AIS, peripheral embolism, or pulmonary embolism) and all-cause mortality at 1-year follow-up. Accounting for patients lost to follow-up, the development dataset comprised 1150 patients with known survival status and 1084 with known thromboembolic status at 1 year.

Our dataset and EHS enrolled patients from hospitals and acute centres across 38 and 35 countries, respectively. Our centres were globally distributed while the Euro Heart Survey was pan-European. Recruitment ran from 1992 to 2006 for the VISTA contributing trials and September 2003 to July 2004 for EHS. The differences in the eligibility criteria of VISTA’s contributing trials and EHS reflect their stroke- and AF-centric aims, respectively. Our less-stringent screening filters were a deliberate attempt to enhance the applicability of CHA_2_DS_2_-VASc to a broad subset of AF patients, not least because of the already stringent inclusion and exclusion criteria of VISTA’s contributing trials relative to that of the observational studies comprising EHS. Finally, the differences in outcome variables reflect our focus on secondary prevention as opposed to primary prevention.

### 2.12. Statistical Analysis

#### 2.12.1. Overview

We will validate the predictive performance of CHA_2_DS_2_-VASc for 90-day recurrence and secondary outcomes in the AF patient cohort by examining discrimination (AUC), calibration (calibration curves and Brier scores), and clinical utility (decision curve analysis). For CPR updating, we will use logistic regression (LR), extreme gradient boosting (XGBoost), and multilayer perceptron (MLP) models, with training and tuning performed through 5 × 2 nested cross-validation. The MLP model will employ transfer learning to incorporate information from the non-AF cohort. Model performance will be assessed on a hold-out test set using AUC, calibration curves, F1 score, and accuracy. We will utilise Shapley additive explanations (SHAP) for model interpretation and to construct two CPRs per model: one based on SHAP main effects and another incorporating both SHAP main and interaction effects. These CPRs will be evaluated against each other, CHA_2_DS_2_-VASc, and default strategies, with test tradeoff analysis to balance ease-of-use with clinical utility.

#### 2.12.2. Data Preprocessing

##### Missing Data Management Imputation

Routine missing data diagnostics will first be conducted: the proportion of missing values for each variable will be reported and visually illustrated by means of a missing data heatmap. The correlation of missingness between variables will be reported pairwise and a dendrogram will be used to provide a comprehensive visual representation of pairwise comparisons.

Multiple Imputation by Chained Equations using Random Forests (MICE RF) will be implemented to impute missing values, based on the algorithm proposed by Doove et al. [93]. Multiple imputation is today’s state-of-the-art solution for handling missing data and, by incorporating a recursive partitioning technique in the MICE framework, this method can fit interactions, non-linear relationships, and complex distributions within the data. As a result, more precise and reliable imputations are achievable as the gains in preserving interaction effects have been found to outweigh the somewhat higher biases for main effects [93], a tradeoff we consider justified given our presumption of interaction effects. Importantly, the missing data mechanisms assumed by MICE (missing completely at random [MCAR] or missing at random [MAR]) are almost impossible to definitively establish [94] and the missing data diagnostics mentioned above are suited only to intuit the plausibility of MCAR. To alleviate this issue, we will include available auxiliary variables in the imputation model that are predictive of missingness but will not be used in the data analysis stages, namely hyperlipidaemia, smoking status, and 30-day NIHSS. Incorporating auxiliary variables has been shown to make the MAR assumption more plausible [95,96], all the while posing little risk to the precision or bias of estimates.

To avoid information leakage, imputation will be performed strictly within each training fold of the inner and outer cross-validation loops, and never on the full dataset before splitting. Any necessary scaling or encoding of continuous variables (e.g., NIHSS, mRS, OTT, VKA days) will likewise occur within-fold. A total of 10 imputation cycles will be performed before aggregating the respective models’ outputs (i.e., computing the mean for continuous variables and selecting the mode for binary variables), with 100 trees per RF model. The constituent features of CHA_2_DS_2_-VASc will be imputed and the score calculated after imputation is complete, which has been reported to be the more appropriate approach for sumscores constructed from relatively few variables [94]. Of note, MICE RF will be performed separately for AF and non-AF cohorts because the validity of later analyses depends on the non-AF cohort having in no capacity shaped the models trained on the AF cohort.

Although missing data ubiquitously occur in RCTs, VISTA did not record how contributing trials handled missing data.

##### Imputation Diagnostics

Convergence of the MICE RF algorithm will be examined using trace plots of the mean and variance of the imputed values across iterations, ensuring that these metrics stabilise over the 10 imputation cycles. The distributions of observed and imputed values will be compared using density plots and histograms, visualising whether the imputed data are faithful to the observed data’s distributional characteristics. Finally, despite the fact that the missing data assumptions of complete case analysis (CCA) are stronger than those of MICE [97], we will perform a sensitivity analysis by comparing the results of our primary analyses on both the imputed and complete-case datasets.

##### Variable Encoding

Comorbid features, tobacco use, IVT, recurrence, and mortality will be encoded as: absent = 0 and present = 1. Sex will be encoded as: male = 0 and female = 1, as stipulated by CHA_2_DS_2_-VASc. The number of days of VKA use over 90-day follow-up will be treated as a continuous variable.

Age requires a more nuanced approach to preserve the structure of the existing CPR. For the validation analyses, age will be encoded as usual: age < 65 years = 0, 65–74 years = 1, and ≥75 = 2. For the updating analyses, the age categories (<65, 65–74, ≥75 years) will be one-hot encoded. This approach involves creating three binary variables and thereby avoids the introduction of an artificial ordinal scale that could imply a linear progression of risk across age groups. As such, it respects the non-linear risk increments that have been reported with advancing age and acknowledged by the existing CPR. Age categories will be fixed, and, in logistic regression, one dummy variable will be dropped from each categorical set to avoid the dummy variable trap.

All other variables (mRS, NIHSS, OTT) will be kept in their original format.

#### 2.12.3. Descriptive Statistics

The Shapiro–Wilk test will be performed to test for normality. Continuous and ordinal variables with normal distributions will be reported as means and standard deviations, while non-normally distributed variables will be reported as medians and interquartile ranges. Binary variables will be reported as counts and ratios of valid observations. All descriptive statistics will be provided both with and without imputation of missing values. Comparisons between groups will be made using the independent samples t-test, the Kruskal–Wallis test, and χ^2^ test, as appropriate.

#### 2.12.4. Validation

We will evaluate CHA2DS2-VASc on the entire AF patient dataset using discrimi-nation, calibration, and clinical utility (Table 1).

Discrimination refers to the ability to correctly identify patients with the outcome of interest (i.e., 90-day recurrence, secondary outcomes), while calibration assesses the alignment between predicted and observed probabilities of outcomes. Discriminative performance will be reported using the AUC (equivalent to the concordance probability or ‘C-statistic’ for binary outcomes) with 95% CIs computed using 1000 bootstrap replicates.

Calibration will be investigated graphically using calibration curves. Calibration curves will also be used as an alternative to ROC curves, as they illustrate true and false positive rates across different risk thresholds and thus help visualise discrimination. Recent recommendations [98], including within the TRIPOD Explanation and Elaboration document [99], suggested that classification curves are preferable to ROC curves for visualising discrimination. Calibration curves will be constructed using loess smoothing. In addition, we will report calibration-in-the-large (CITL) and the expected/observed (E/O) event ratio.

Additionally, F1 score, accuracy, precision, sensitivity, and specificity will be reported. Youden index, positive predictive value (PPV), negative predictive value (NPR), positive likelihood ratio (PLR), and negative likelihood ratio (NLR) will be supplementarily provided.

If a model demonstrates better discrimination but worse calibration than the other, or vice versa, deciding how to rank them would be rather arbitrary [100]. This issue will be addressed later on by investigating clinical utility using decision curve analysis. See “CPR Evaluation”.

Importantly, we will also report the discrimination, calibration, and clinical utility of CHA_2_DS_2_-VASc on the non-AF cohort, deploying the same protocol as for AF patients.

**Table 1:** Validation analyses.

Questions	Hypotheses	Outcome Measures	Sampling Plan (*N*, Power Analyses)	Analysis Plan	Interpretation Given to Different Outcomes
Primary: How well does CHA_2_DS_2_-VASc capture 90-day AFAIS recurrence risk in terms of discrimination, calibration, and clinical utility? (Clinical utility performed against updated CPRs, see Tables 3 and 4.) Secondary: How well does CHA_2_DS_2_-VASc capture: 7-day recurrence, 7- and 90-day HT, 90-day decline in functional status, and 90-day all-cause mortality? Exploratory: How well does CHA_2_DS_2_-VASc capture 90-day recurrence and secondary outcomes for non-AF AIS patients?	Primary: Discrimination of CHA_2_DS_2_-VASc for 90-day AFAIS recurrence risk is unsatisfactory (AUC < 0.6), at worst, and modest (<0.7), at best. Calibration for 90-day recurrence also leaves much room for improvement. Secondary: As above.	Discrimination assessed using AUC and calibration curves. Calibration assessed using calibration curves, slopes, and Brier scores. Additional metrics: F1 score, accuracy, precision, sensitivity, specificity, Youden index, PPV, NPR, PLR, NLR.	Primary: A minimum of 680 patients deemed necessary, see Appendix S5.3 in Supplementary Materials file S3. Entire AF dataset will be used, comprising 2763 AFAIS patients. Secondary: As above. Exploratory: Entire non-AF dataset will be used, comprising 7809 AIS patients.	Compute AUCs with 95% CIs using 1000 bootstrap replicates. Produce calibration curves (and report calibration slopes) to illustrate calibration. Calibration curves also serve as alternative to ROC curves, as they illustrate true and false positive rates across different risk thresholds and thus help visualise discrimination. Compute Brier scores. Compute additional metrics.	Discrimination for each outcome will be classified as follows [101]: AUC of 0.81–0.90 = good, 0.71–0.80= fair, 0.61–0.70 = modest, and 0.51–0.60 = poor. In practice, calibration is more vulnerable to geographic and temporal heterogeneity than discrimination [102,103,104,105]. We thus stress that calibration is at least as important as discrimination [77,102]. Clinical utility via DCA will go beyond discrimination and calibration, considering them both at the same time [106], as well as individual preferences.

#### 2.12.5. Updating

##### Models

LR, XGBoost, and MLP classifier models will be used to establish the updated CPR. LR and XGBoost will power our primary analyses, and MLP our secondary analyses.

##### Primary Analysis

LR will serve as our baseline model, which is in keeping with how CPRs have traditionally been developed and reflects the fact that regression analysis tends to perform remarkably well [107]. As a matter of fact, the predictive performance of ML models in the context of CPMs has been reported to be non-superior to that of LR when comparisons had low risk of bias [60]. As an interpretable model, LR will enable us to unequivocally ascertain whether a feature has a positive or negative impact on the probability of an outcome by virtue of the odds ratio (OR).

XGBoost will be used to free our primary analysis from the linear separability constraint of LR. As a decision tree ensemble method, XGBoost iteratively fits the data to minimise residuals by adding trees that focus on areas where previous trees underperformed. By combining the predictions of multiple trees, XGBoost can capture complex, non-linear interactions, making it particularly effective for irregular decision boundaries where linear models fall short.

##### Secondary Analysis

DL will be used to leverage the non-AF cohort data. A transfer learning strategy will be adopted: the MLP (a multilayered feed-forward neural network) will be pretrained on the non-AF dataset, and the best-performing pretrained model will be transferred to the AF dataset for fine-tuning. In other words, the model will initially learn from the larger dataset and then refine its parameters downstream on the target dataset, enhancing its predictive performance for the latter. This technique exploits the layer-wise modular architecture of neural networks, that is, the fact a network can be constructed by removing all layers after a particular layer and appending a new connected layer with a different configuration of neurons and initial weights. As a result, the MLP will have learnt from 10,572 AIS patients, all the while prioritising the 2763 with AF. Indeed, the pretraining paradigm has become dominant because it has enabled the use of neural networks in small datasets, where it would not be cautious to train models from scratch [108]. Generally, the more similarity between the datasets, the more benefits conferred by transfer learning, including higher asymptotic accuracy, enhanced generalisability, and accelerated convergence. We consider our situation well-suited to this technique: the features of CHA_2_DS_2_-VASc are known to increase risk of AIS in patients without AF [40,71] and CHA_2_DS_2_-VASc does not incorporate AF-related parameters that impact AIS risk, such as duration of AF and left atrium/left atrial appendage (LAA) size, function, and morphology [101]. It has even been reported that CHA_2_DS_2_-VASc may be equally discriminative in the absence of AF [40].

Of course, no preprocessing parameters or summary statistics will be carried over from the non-AF to AF datasets. Pretraining (including its internal validation) will use only non-AF data, and during AF model development, all preprocessing will be fit within AF training folds only, with the AF hold-out test set untouched until final evaluation.

#### 2.12.6. Regularisation

##### Multicollinearity

Multicollinearity is a common phenomenon in clinical prediction modelling and means that the data offers limited information on how the explained variance in the outcome should be distributed over the collinear predictors [109]. Although this is generally not considered problematic with regard to predictive performance [110], we will report the variation inflation factor (VIF) to acknowledge that there may not be just one way, but multiple ways, to construct the CPR that are equally valid in our data [111]. VIF > 5 will be considered indicative of significant multicollinearity.

##### Regularisation Techniques

LR will be instantiated with L2 regularisation (ridge regression). L2 introduces a penalty proportional to the sum of the squared coefficients to the loss function. This shrinks the coefficients of less important features and discourages an overly complex model, thereby stabilising the estimates and improving generalisability.

For XGBoost, we will use built-in tree-specific regularisation hyperparameters to constrain model complexity and mitigate overfitting. See “Hyperparameter Tuning” below.

MLP will be instantiated with L2 (weight decay) and dropout, complementary regularisation techniques that limit the magnitude of weights and prevent complex coadaptation of neurons, respectively. As a result, the network will be prevented from becoming too complex or overly dependent on specific neurons and encouraged to learn more generalised patterns.

##### Hyperparameter Tuning

Each model class is characterised by a unique suite of hyperparameters that play a pivotal role in shaping performance. Manual trial-and-error to configure these values is computationally irreproducible and prone to bias [112]. To avoid obfuscating our models’ internal architectures, we will automate hyperparameter optimisation using grid search within the predefined hyperparameter search spaces tabulated below. The search spaces are intentionally small, as limiting the number of adjustable elements is another means to impose constraints on complexity (Table 2).

##### Model Building LR and XGBoost

We will instantiate LR with L2 regularisation and default hyperparameters (i.e., C = 1, max_iter = 100, solver = ‘lbfgs’), and XGBoost with default hyperparameters (i.e., n_estimators = 100, learning_rate = 0.1, max_depth = 3, objective = ‘binary:logistic’).

**Figure 1:**
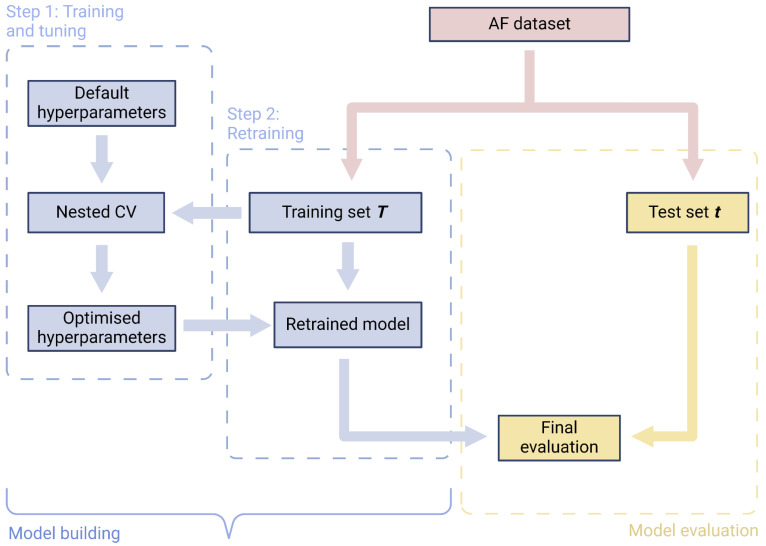
Workflow for model building and evaluation.

The AF dataset will be randomly split into two parts: training (***T***, 75%) and test sets (***t***, 25%), ensuring that the distribution of outcomes is preserved across both sets. LR and XGBoost models will each be trained, hyperparameter-tuned, and retrained on the training set (***T***), while the independent test set (***t***) will be withheld for final evaluation (Figure 1).

**Figure 2:**
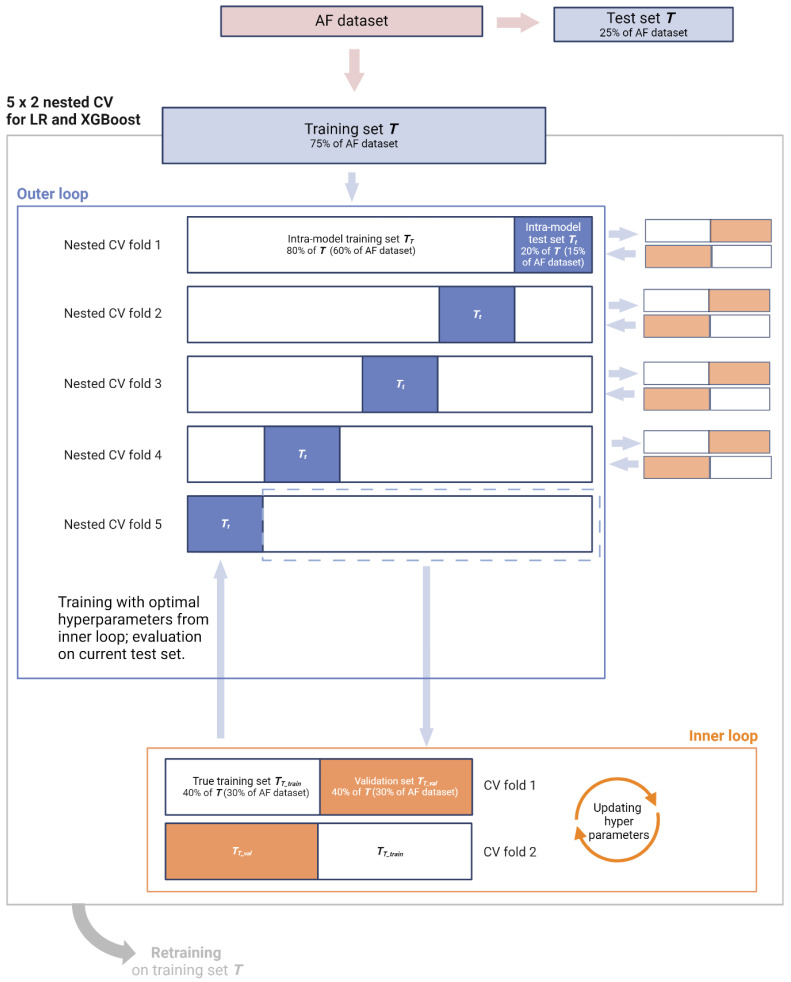
Overview of 5 × 2 nested cross-validation.

To achieve robust final models, we will employ nested cross-validation (CV) for training and hyperparameter tuning. Nested CV is particularly effective for small to moderately sized datasets because it promotes generalisability by ensuring that the hyperparameter tuning process does not contaminate the internal evaluation phase [113]. To this end, a 5 × 2 setup will be utilised, consisting of 5-fold CV in the outer loop and 2-fold CV in the inner loop (Figure 2). The set ***T*** will first be split into five equal parts. In each fold of the outer loop, one part (20% of ***T***) will serve as the intra-model test set ***T_t_*** while the remaining 80% will serve as the intra-model training set ***T_T_***. Each outer loop iteration will be subjected to an inner loop that further splits ***T_T_*** into two equal parts, using one as the true training set ***T_T_train_*** and the other as the validation set ***T_T_val_***, and alternating them over the two folds. The primary selection metric in the inner CV will be log loss (binary cross-entropy), with ties broken by calibration performance (Brier score).

Instantiating the models using the hyperparameters determined by the above procedure, we will retrain them in order to leverage all patients in ***T*.** The resulting models will be considered final and subjected to evaluation on ***t*** in the next section (see “Model Evaluation”). At this stage, training performance will be recorded. Specifically, we will report the area under the receiver operating characteristic curve (AUC), F1 score, accuracy, precision, sensitivity, and specificity. Coefficients and ORs (exponentiated coefficients) with 95% confidence intervals (CIs) will be reported for LR, and the hierarchy of predictor variables in descending order of gain (its internal feature importance metric) will be provided for XGBoost. Individual decision trees within the XGBoost model will supplementarily be made available.

##### MLP

MLP will be instantiated with default hyperparameters (i.e., optimizer = Adam(learning_rate = 0.01), loss = ‘binary_crossentropy’, metrics = [‘AUC’]), units = 32, hidden_layers = 2, activation = ‘relu’, kernel_regularizer = l2(0.01), dropout = 0.2 for the pretrained model; and the best hyperparameters found during pretraining for the fine-tuned model, with the exception of learning_rate = 0.00001).

Unlike the AF dataset, the non-AF dataset will not undergo random splitting. Rather, the MLP will be trained, hyperparameter-tuned, and retrained on the whole non-AF dataset to yield the so-called pretrained model. As above, 5 × 2 nested CV will be implemented and the best hyperparameters will be used to retrain the model on all non-AF patients.

The pretrained model will then be evaluated (AUC, F1 score, precision, sensitivity, specificity) and its last layer(s) will be removed, which are expected to be the more task-specific ones (i.e., non-AF patient-centric). These layers will be replaced by new, naive layers that culminate in an output layer with two neurons, given that we are performing probabilistic binary classification. The resulting neural network will be trained on ***T***, the training set of the AF dataset. Two strategies will be tried: fine-tuning of all layers, including pretrained ones (albeit with very low learning rates), and freezing of pretrained layers, with training confined to the newly added layers. In either case, the training phase will again employ 5 × 2 CV and the fine-tuned model will be retrained on ***T*** as a last step. Early stopping will be implemented with a patience parameter of 10 epochs, monitored on the validation loss during training.

Training performance will be reported using AUC, F1 score, accuracy, precision, sensitivity, and specificity. AUC, F1 score, accuracy, precision, and sensitivity will be used to inform whether the purely fine-tuned or the frozen model will be retained for evaluation on ***t***.

For reproducibility, matched random seeds will be set and reported across LR, XGBoost, and MLP models.

##### Model Evaluation Test Set

The test set ***t***, disjointed from the set used in model building, will be used to evaluate and compare the performance of our final LR, XGBoost, and MLP models. As a randomly sampled subset of the development cohort, this test set will serve as an internal validation tool. Differences between test and training performance will also be reported, as this will be informative on the degree of overfitting to the training set [60].

The discriminative performance of each model will be reported using the AUC. AUCs will be reported with 95% CIs computed as the mean of 1000 bootstrap replicates ± 1.96 standard deviations. Since the AUCs will be derived from the same test set, we will also use DeLong’s method, a non-parametric approach to constructing CIs that accounts for implicit correlation between the underlying ROC curves [114].

Calibration will be investigated graphically using calibration curves. Calibration slopes and Brier scores will also be reported.

Additionally, F1 score, accuracy, precision, sensitivity, and specificity will be reported. Youden index, positive predictive value (PPV), negative predictive value (NPR), positive likelihood ratio (PLR), and negative likelihood ratio (NLR) will be supplementarily provided.

Coefficients and ORs will be tabulated for LR, and the hierarchy of predictor variables in descending order of gain will be provided for XGBoost. Individual decision trees within the XGBoost model will supplementarily be made available.

##### Further Evalution of Primary Analysis Models

We will also investigate the discrimination and calibration of the LR and XGBoost models on the non-AF cohort. This will fortify comparative exploration of performance by leveraging a large, related dataset that was heretofore unseen by the two primary analysis models. To clarify, the reason the MLP model is excluded from this step is because the non-AF cohort was implicated in its training.

##### Model Interpretation

Understanding why a model makes certain predictions is often as crucial as its overall performance [115]. To shed light on the internal logic behind our models’ predictions of 90-day recurrence, we will employ Shapley additive explanations (SHAP). SHAP is a model-agnostic framework to explain individual predictions and currently a dominant approach in interpretable ML (IML) [116]. In brief, SHAP values quantify feature importance by treating the prediction process as a cooperative game among the predictor variables. An attractive property of SHAP is that it provides both local explanations and insights into global model behaviour [117]. Specifically, the mean absolute SHAP value of a predictor variable serves as a heuristic for assessing its importance to the model’s global performance [115,118]. Furthermore, formal analysis of global feature importance is possible via SAGE (Shapley Additive Global Importance), a performance-based extension of SHAP [118].

The best explanation of a simple model is the model itself; however, using the original model as its own explanation is infeasible with models such as XGBoost and MLP due to the complexity of their internal informational architecture [115,119]. Although it is inherently interpretable, we will use SHAP and SAGE for LR too. This will ensure a consistent framework for reporting feature importance across models. Of note, the fact that LR is our baseline model is itself an ante hoc IML method [120].

##### Local Interpretation

For each model, we will select 20 predictions to individually explain. Of these, 10 will be randomly selected and 10 will be selected as cases of special interest (i.e., borderline and misclassified instances). Matched random seeds will be used for the former. For each prediction, the SHAP value of each feature will be reported. SHAP force plots will be provided to visually convey the contribution of each feature to the prediction. SHAP interaction values—calculated pairwise as the difference between the SHAP value for feature *i* when *j* is present, and the SHAP value for feature *i* when *j* is absent—will also be reported to quantify local interactions.

##### Global Interpretation

To summarise the average impact of each feature on our models’ outputs across the test set, we will calculate mean absolute SHAP values. The absolute mean will also be taken across SHAP interaction values. SHAP summary plots will be provided: bar plots to depict mean absolute SHAP values, and beeswarm plots to depict the distribution of local SHAP values in order to intuitively understand variability. We will also provide 8 × 8 beeswarm plots which depict interaction effects in the off-diagonal entries of the feature grid, 28 of which are unique owing to the symmetry of SHAP interaction values.

SAGE values will be reported with regard to the AUC and graphically represented using bar plots. Unlike SHAP, the SAGE algorithm readily provides the standard error of each feature’s importance. These will be utilised to gauge the stability of the importance estimates and facilitate their comparison.

##### CPR Development

SHAP will be used to ‘translate’ model insights into CPRs. Mean—and not mean *absolute*—SHAP values will be used in order to preserve information on the directionality of feature importance. The decile rank of each of the eight mean SHAP values from each model will be calculated and a dot plot will be provided to visualise each feature’s mean SHAP value relative to others. (We speak of eight mean SHAP values because we have eight predictor variables corresponding to the features of CHA_2_DS_2_-VASc, namely five comorbid items, sex, and *two* age categories [65–74 years and ≥75 years]). The 25th, 50th, and 75th percentiles of the eight mean SHAP values will be computed. We will subsequently rank the features from most to least important and sort them into three tiers: (1) the lower tier grouping features with mean SHAP values below the 25th percentile, (2) the middle tier grouping features with mean SHAP values between the 25th and 75th percentiles, and (3) the upper tier grouping features with mean SHAP values above the 75th percentile. Features belonging to the lower, middle, and upper tiers will be assigned one, two, and three points, respectively. The purpose of a larger middle tier is to avoid over- or understating the importance of features by promoting stability over frequent changes with minor shifts in model parameters. In the rather unlikely event that one or more features will have negative mean SHAP values and local SHAP value distributions that are consistently left of zero on the beeswarm plots, zero points will be assigned, because their inclusion would degrade 90-day recurrence predictive performance. Ties at quantile cut-points will be resolved conservatively by assigning them to the higher category (e.g., features tied at the 75th percentile will enter the upper tier). The resulting CPRs will be summarised in tabular form and given an acronym in likeness to CHA_2_DS_2_-VASc.

If the dot plot suggests that the distribution of mean SHAP values is very skewed or concentrated, the above-defined tiers will probably inadequately capture features of lower, intermediate, and higher importance. In this eventuality, we will tailor tier boundaries using quantile-based binning. After adapting the tier setup to the observed clusters, we will define lower, middle, and upper tiers whose features will be assigned one, two, and three points, respectively.

Each of the CPRs will subsequently be augmented by integrating interaction effects. This purports to better accommodate for the fact that a given feature may be more impactful in the presence of one of its counterparts. Mean (and not mean *absolute*) SHAP interaction values will be used in order to distinguish between synergistic and redundant interactions. The decile rank of each of the twenty-eight mean SHAP interaction values from each model will be calculated. A dot plot will be provided to visualise each feature-pair’s mean SHAP interaction value relative to other pairs. (We speak of twenty-eight mean SHAP interaction values because there are eight features, and (*i*, *j*)- and (*j*, *i*)-values capture the same interaction effect, while (*k*, *k*)-values capture main effects and were the object of the above paragraph). Dealing with a large number of feature-pairs, we will use stringent percentiles in selecting interaction effects for inclusion. The 90th percentile of the sample of twenty-eight mean SHAP interaction values will be computed. Feature-pairs with mean SHAP values above this threshold will be selected as the most important synergisms. Feature-pair selection will also incorporate domain knowledge, as some feature-pairs have well-established synergisms that may merit inclusion even if they fall somewhat short of the 90th percentile cutoff. Examples include CHF and age [121], hypertension and age [122], and hypertension and DM [123]. Selected feature-pairs will be integrated into the CPRs as accompanying rules: ‘if both feature *i* and *j* of the pair are present in the patient, add one additional point to the patient’s total score’. To mitigate overfitting, we will cap the number of points that can be added to accommodate synergisms to three. We will further cap the number of interaction pairs retained at a maximum of three. The augmented CPRs will be summarised in tabular form and their acronyms labelled with an asterisk (e.g., CHA_2_DS_2_-VASc*). A worked example of the CPR development process here described can be found in Appendix S6 in Supplementary Materials file S3.

Both non-augmented and augmented CPRs will undergo evaluation. See “CPR Evaluation”.

##### CPR Evaluation

We will evaluate the CPRs on the test set ***t*** using discrimination, calibration, and clinical utility. The same methodology previously applied to CHA_2_DS_2_-VASc will be used to assess the discrimination and calibration of the newly developed CPRs. See Section 2.12.4. Clinical utility will be measured using net benefit (NB) via decision curve analysis (DCA). Although a CPR with better discrimination and calibration should, in theory, better guide clinical management, discrimination and calibration cannot determine whether a CPR aids decision making or which of several CPRs leads to better decisions. This is especially true when some CPRs have better discrimination and others better calibration. The shortcomings of conventional measures of performance are due to the fact that they do not incorporate information on consequences. DCA incorporates consequences by considering the tradeoffs between the benefits of true positives and the harms of false positives across a range of risk thresholds that reflect a spectrum of possible patient and clinician preferences [100]. It achieves this by calculating the NB for each risk threshold (*pt*), which is defined as the number of true positives corrected for the number of false positives weighted by the odds of the risk threshold, all divided by the sample size (*N*) [124]. This is equivalent to the proportion of true positives in the absence of false positives (i.e., perfect specificity). To effectively use CPRs in clinical management, it is necessary to specify the risk thresholds at which intervention would be warranted. For early secondary prevention of AFAIS, oral anticoagulation is the treatment, and risk thresholds within 0.01—0.20 are reasonable. We will thus calculate the true positives and false positives for each CPR across the 0.01–0.20 risk threshold range and compute NB using the following formula:

$$NB=\frac{\;\mathrm{True}\;\mathrm{positives}\;}{N}-\frac{\;\mathrm{False}\;\mathrm{positives}\;}{N}\left(\frac{p_{t}}{1-p_{t}}\right).\;$$

NB will be plotted on the *y*-axis against risk threshold on the *x*-axis to yield a decision curve for each CPR. These curves, including the one for CHA_2_DS_2_-VASc, will be presented in a single figure to facilitate comparison. Additionally, we will plot lines representing the default strategies of ‘treat all’ and ‘treat none’, corresponding to scenarios where all or no patients are assumed to suffer 90-day recurrence, respectively. Incidentally, these lines will intersect at the observed prevalence of 90-day recurrence in our test set. A CPR will be considered clinically useful at a given risk threshold only if its NB exceeds that of both default strategies. Conversely, if a CPR’s NB is lower than either default strategy, it will be deemed clinically harmful. Our initial analysis will focus on determining whether the CPRs have higher NBs than the default strategies across the selected risk thresholds. We will then proceed to comparing the CPRs, ranking them based on descending NB, and identifying the three CPRs with the highest clinical utility (Table 3 and Table 4).

If an augmented CPR ranks among the three highest, we will perform test tradeoff analysis to compare it with the closest-ranking non-augmented CPR. The test tradeoff, calculated as Δ*NB*, represents the minimum number of additional tests per true positive required to justify the augmented CPR’s use, considering its added cost [124]. Of course, in our case, cost is not financial, but the additional time and reduced convenience associated with augmented CPRs, which include accompanying rules and are thereby more unwieldy than their non-augmented counterparts. If the NB differences across thresholds are small, the test tradeoff will be large, suggesting that the simpler CPR may be better, all things considered. After all, CHA_2_DS_2_-VASc owes its widespread adoption to its parsimonious, back-of-the-envelope nature. For clarity, we will report test tradeoffs in terms of both true positives and true negatives. The same approach will be applied to CPRs developed using MLP, where the test tradeoff will help decide whether the increased NB justifies the involvement of a less interpretable model. Ultimately, the purpose of reporting test tradeoffs will be to highlight which CPRs—if any—would be conveniently deployable without sacrificing significant clinical utility, and *not* to dismiss higher cost CPRs from future consideration.

Finally, we will also report the clinical utility of the LR- and XGBoost-powered CPRs and CHA_2_DS_2_-VASc on the non-AF cohort. The purpose of this is to establish whether they remain better than the default strategies when exposed to a large set of patients that non-randomly differs from the training dataset.

Table 3 and Table 4 should appear after Section “CPR Evaluation”.

##### External Validation

The performance of CPRs often markedly declines when applied to independent datasets [125]. Regular, robust external validations are therefore indispensable to accurately assess a CPR’s generalisability over time and space. While some journals require an external validation section in CPR studies, the legitimacy of this requirement has been called into question [77]. Mandating a single ‘snapshot’ validation may inadvertently lead to selective reporting of favourable results from a specific setting, rather than promoting a thorough evaluation across diverse populations. This may prove particularly counterproductive when ML and DL models are involved, as these models can harbour embedded biases.

Given these considerations, we argue that the external validation of our CPRs deserves its own dedicated study, complete with a preregistered protocol that includes tailored power analyses and strategies to quantify patient heterogeneity. We hereby commit to a follow-up study that will not only strengthen the credibility of our CPRs but also contribute to advancing best practices in CPR research. External validation is not merely an item on a checklist, but a rigorous investigation in its own right, requiring careful planning and registration before execution.

##### Fairness

To enhance the fairness of our CPRs, we will explore how porous they are to any entrenched gender and age biases.

##### Gender Bias

There are concerns that CHA_2_DS_2_-VASc may overestimate stroke risk in women, particularly younger women and those who have no other risk factors [126]. We will supplementarily report discrimination, calibration, and DCA separately for male and female patients in order to determine whether the gender adjustment of our CPRs is warranted.

##### Age Bias

By treating age as a categorical and not a continuous variable, our CPRs may oversimplify the relationship between ageing, comorbidities, and AIS risk. After all, AIS risk does not abruptly rise on a patient’s 65th or 75th birthday. We will supplementarily report discrimination, calibration, and DCA separately for <65-, 65–74-, and ≥75-year-old patients to determine whether the age adjustments of our CPRs are warranted. DCA will also be performed for patients within the highest and lowest age deciles so as to compare the CPRs against the default strategies and identify any potential under- or over-treatment. This will be performed across all patients and as well as separately for male and female patients. Age-stratified analyses will be conducted across all patients as well as sub-stratified by gender.

##### Comment on Ethnic and Socioeconomic Biases

CHA_2_DS_2_-VASc was predominantly developed and validated using data from white European populations. It is encouraging that VISTA’s contributing trials included centres from diverse parts of Europe, North America, South America, Asia, and Oceania. However, we take notice of the limited representation of African populations, as only South Africa was included from the African continent. The underrepresentation of African populations is a serious obstacle to the global applicability of CPRs, and TRIPOD-P explicitly calls for the contribution of individuals with CPR expertise from Africa, as well as Asia, Central America, and South America [70].

VISTA did not record patient race and socioeconomic status (SES), meaning we will not be able to perform race- and SES-stratified analyses, nor report on the proportion of African American and Latino American patients, for example. It has been found that both African American and Latino American patients with CHA_2_DS_2_-VASc scores of two or more were less likely to receive NOACs than white patients [127]. While the disparity between Latino American and white patients did not persist after adjusting for SES, the difference between black and white patients remained significant. This highlights the critical need for population-based research cohorts for fine-tuning CPRs to existing health disparities [128].

## Figures and Tables

**Table 2:** Search spaces for hyperparameter tuning.

Model		Hyperparameter	Values
LR		C (L2 regularisation strength)	[0.01, 0.1, 1, 10, 100]
		max_iter	[100, 200, 500]
XGBoost		n_estimators	[50, 100, 200]
		learning_rate	[0.01, 0.1, 0.2]
		max_depth	[3, 5, 7, 9]
MLP	Pretraining	units (hidden layer sizes)	[32, 64, 128, 256, 512]
		hidden_layers	[1, 2, 3, 4]
		activation	[‘relu’, ‘tanh’]
		learning_rate	[0.0001, 0.001, 0.01]
		kernel_regularizer = l2()	[0.000001, 0.00001, 0.0001, 0.001, 0.01, 0.1]
		dropout (dropout rate)	[0.1, 0.2, 0.3, 0.4, 0.5]
	Fine-tuning	units (hidden layer sizes)	[32, 64, 128, 256, 512]
		hidden_layers	[1, 2, 3, 4]
		activation	[‘relu’, ‘tanh’]
		learning_rate	[0.000001, 0.00001, 0.0001]
		kernel_regularizer = l2()	[0.000001, 0.00001, 0.0001, 0.001, 0.01, 0.1]
		dropout	[0.1, 0.2, 0.3, 0.4, 0.5]
		Freezing layers	Custom implementation

**Table 3:** Updating analyses; primary.

Questions	Hypotheses	Outcome Measures	Sampling Plan (*N*, Power Analyses)	Analysis Plan	Interpretation Given to Different Outcomes
Primary: Does the acute context of AFAIS, and the importance of the index event, call for a CPR bespoke to early secondary prevention? In other words, are the main effects of the constituent features of CHA_2_DS_2_-VASc different post-stroke? Besides main effects, are there synergistic or other non-linear interaction effects between stroke history and the constituent features of CHA_2_DS_2_-VASc that can further improve prediction of 90-day AFAIS recurrence? Secondary: How do CPRs trained to capture 90-day AFAIS recurrence perform with regard to secondary outcomes (7-day recurrence, 7- and 90-day HT, 90-day decline in functional status, and 90-day all-cause mortality)? Exploratory: How do CPRs trained to capture 90-day AFAIS recurrence perform with regard to recurrence among non-AF AIS patients?	Primary: LR and XGBoost can capture the main effects of the constituent features of CHA_2_DS_2_-VASc in the context of secondary prevention. XGBoost is also equipped to capture complex functional relationships between the features. The outputs of these models can be employed to construct CPRs that outperform CHA_2_DS_2_-VASc for predicting 90-day recurrence in AFAIS patients. Specifically, fair (AUC < 0.8) to good (< 0.9) discrimination can be achieved for this high-risk group, alongside improved calibration and clinical utility relative to CHA_2_DS_2_-VASc. Secondary: The resulting CPRs also outperform CHA_2_DS_2_-VASc for predicting secondary outcomes.	LR and XGBoost models: Discrimination assessed using AUC and calibration curves. Calibration assessed using calibration curves, slopes, and Brier scores. Additional metrics: F1 score, accuracy, precision, sensitivity, specificity, Youden index, PPV, NPR, PLR, NLR. Resulting CPRs: Discrimination assessed using AUC and calibration curves. Calibration assessed using calibration curves, slopes, and Brier scores. Clinical utility assessed via DCA relative to default strategies as well as CHA_2_DS_2_-VASc. Test tradeoff analysis will be used to balance ease-of-use with clinical utility.	Primary: LR—680 patients deemed necessary, see Appendix S5.3 in Supplementary Materials file S3. XGBoost—learning curve analysis and KL divergence will afford post hoc insight on suitability of sample size. 75% of AF dataset will be used, comprising 2072 AFAIS patients. Remaining 25% (691 patients) will be withheld as test set. Secondary: Entire AF dataset will be used, comprising 2763 AFAIS patients. Exploratory: Entire non-AF dataset will be used as supplementary test set, comprising 7809 AIS patients.	Models will be instantiated (LR with L2 regularisation and XGBoost with default hyperparameters) on AF dataset, randomly split into training (***T***, 75%) and test sets (***t***, 25%), preserving distribution of outcomes. Nested cross-validation (5 × 2) will be employed for training and hyperparameter tuning. After hyperparameters are optimised, final models will be retrained using the entire training set. The test set ***t*** will then be used for evaluation of final LR and XGBoost models, focusing on discrimination and calibration. Feature importance will be reported via coefficients and odds ratios (ORs) for LR and feature gain for XGBoost. To derive CPRs from these models, SHAP will quantify both main effects and interaction effects of predictor variables. Main effects, represented by mean SHAP values, will be used to rank and assign scores to each predictor, enabling the creation of interpretable and clinically applicable CPRs. Interaction effects, captured through SHAP interaction values, will further refine our CPRs by identifying significant synergistic or redundant relationships between predictors. These interactions will be incorporated into so-called ‘augmented CPRs’ in the form of accompanying rules. Both non-augmented and augmented CPRs will be evaluated on the test set ***t*** in terms of discrimination, calibration, and clinical utility. Clinical utility will be measured using NB via DCA, plotting NB against risk thresholds within the 0.01–0.20 range. The CPRs will be compared on the basis of clinical utility to one another, CHA_2_DS_2_-VASc, and default strategies (‘treat all’ and ‘treat none’). Test tradeoff analysis will evaluate whether any incremental benefit in clinical utility observed with augmented CPRs justifies the added complexity of incorporating accompanying rules. Secondary: CPRs (and not models) will be evaluated on the entire AF dataset for each secondary outcome via discrimination, calibration, and clinical utility. Exploratory: CPRs (and not models) will be evaluated on the entire non-AF cohort to assess generalisability and compare their clinical utility when applied to an unseen yet closely related patient population.	Discrimination will be classified as follows [101]: AUC of 0.81–0.90 = good, 0.71–0.80 = fair, 0.61–0.70 = modest, and 0.51–0.60 = poor. Miscalibration is detrimental to medical decision making and poor calibration has been coined the Achilles heel for CPR applicability [102]. As such, we consider at least as important as discrimination and give precedence to clinical utility, which provides a more comprehensive evaluation by integrating both discrimination and calibration when interpreting the performance of our CPRs. CPRs will be considered clinically useful at a given risk threshold only if their NBs exceed those of both default strategies. Of those deemed useful, we will rank the CPRs (including CHA_2_DS_2_-VASc) based on NB across the 0.01–0.20 risk thresholds. Test tradeoff (∆*NB*) will be interpreted only insofar as spotlighting those CPRs which would be more conveniently deployable without sacrificing significant clinical utility.

**Table 4:** Updating analyses; secondary.

Questions	Hypotheses	Outcome Measures	Sampling Plan (*N*, Power Analyses)	Analysis Plan	Interpretation Given to Different Outcomes
Primary: Does the acute context of AFAIS, and the importance of the index event, call for a CPR bespoke to early secondary prevention? In other words, are the main effects of the constituent features of CHA_2_DS_2_-VASc different post-stroke? Besides main effects, are there synergistic or other non-linear interaction effects between stroke history and the constituent features of CHA_2_DS_2_-VASc that can further improve prediction of 90-day AFAIS recurrence? Secondary: How do CPRs trained to capture 90-day AFAIS recurrence perform with regard to secondary outcomes (7-day recurrence, 7- and 90-day HT, 90-day decline in functional status, and 90-day all-cause mortality)?	Primary: MLP can capture the main effects of the constituent features of CHA_2_DS_2_-VASc in the context of secondary prevention, as well as complex functional relationships between stroke history and the other features. Its output can be employed to construct CPRs that outperform CHA_2_DS_2_-VASc for predicting 90-day recurrence in AFAIS patients. Specifically, fair (AUC <0.8) to good (<0.9) discrimination can be achieved for this high-risk group, alongside improved calibration and clinical utility relative to CHA_2_DS_2_-VASc. Secondary: The resulting CPRs also outperform CHA_2_DS_2_-VASc for predicting secondary outcomes.	See Table 3.	Learning curve analysis and KL divergence will afford post hoc insight on suitability of sample size. Data augmentation via transfer learning is particularly valuable for DL models like MLP, which are very data-hungry. Entire non-AF dataset will be used in pretraining phase, comprising 7809 AIS patients. 75% of AF dataset (***T***) will be used in fine-tuning phase, comprising 2072 AFAIS patients. Remaining 25% of AF dataset (***t***, 691 patients) will be withheld as test set.	The MLP model will leverage information within the non-AF patient dataset during pretraining. Non-AF AIS patients, who were not included in training the LR and XGBoost models—and therefore in no capacity informed the CPRs constructed thus far—may improve CPR generalisability and clinical utility. We contend our use case is well-suited to transfer learning given that the features of CHA_2_DS_2_-VASc are known to increase risk of AIS in patients without AF, and it has been reported to be equally predictive in the absence of AF [40]. MLP will be instantiated using default hyperparameters and undergo training, hyperparameter tuning, and retraining on the entire non-AF dataset to yield the pretrained model. The pretrained model will be evaluated (AUC, F1 score, precision, sensitivity, specificity) and its last layer(s) removed. These will be replaced by new, naive layers that culminate in an output layer with two neurons. The resulting neural network will be trained on the training set ***T***. Two strategies will be tried: fine-tuning and freezing. In either case, the training phase will again employ nested CV (5 × 2) and the fine-tuned model will be retrained on the AF training set. Training performance will be reported using AUC, F1 score, accuracy, precision, sensitivity, and specificity, used to inform whether the purely fine-tuned or the frozen model will be retained for evaluation on the test set ***t***. To derive CPRs from the MLP model’s output, SHAP will quantify main and interaction effects of predictor variables. Main effects, represented by mean SHAP values, will be used to rank and assign scores to each predictor. Interaction effects, captured through SHAP interaction values, will further refine our CPR by identifying significant synergistic or redundant relationships between predictors. These interactions will be incorporated into an ‘augmented CPR’ in the form of accompanying rules. Both non-augmented and augmented CPRs will be evaluated on the test set ***t*** in terms of discrimination, calibration, and clinical utility. The CPRs will be compared on the basis of clinical utility to one another, LR- and XGBoost-informed CPRs, CHA_2_DS_2_-VASc, and default strategies (‘treat all’ and ‘treat none’). Test tradeoff analysis will be performed. Secondary: CPRs (and not the MLP model) will be evaluated on the test set ***t*** for each secondary outcome via discrimination, calibration, and clinical utility.	See Table 3.

## Data Availability

To access the data, interested parties must likewise submit a proposal to the VISTA-Acute Steering Committee. The dataset was previously inaccessible to all authors.

## Supplementary Materials

The following supporting information can be downloaded at: https://www.mdpi.com/article/10.3390/jcm14207327/s1. Supplementary Materials file S1: Collaborators/Membership of the Group/Team Name; Supplementary Materials file S2: TRIPOD+AI checklist; Supplementary Materials file S3: Appendix.
