# Supplementary material for: Secondary Prevention of AFAIS: Deploying Traditional Regression, Machine Learning, and Deep Learning Models to Validate and Update CHA2DS2-VASc for 90-Day Recurrence
Source: J Clin Med. 2025 Oct 16;14(20):7327. doi: 10.3390/jcm14207327 (PMC12565214; doi:10.3390/jcm14207327)
Supplement: Supplementary file 1 [file jcm-14-07327-s001.zip › jcm-3802014-supplementary file S1 (name).pdf]

The VISTA-Acute Collaboration: * K.R. Lees (Chair), A. Alexandrov, P.M. Bath, E. Bluhmki, N. Bornstein, C. Chen, L. Claesson,

J. Curram, S.M. Davis, H-C. Diener, G. Donnan, M. Fisher, M. Ginsberg, B. Gregson, J. Grotta,

W. Hacke, M.G. Hennerici, M. Hommel, M. Kaste (Emeritus), P. Lyden, J. Marler, K. Muir, C. Roffe, R. Sacco, A .Shuaib, P. Teal, N. Venketasubramanian, N.G. Wahlgren, and S. Warach
