# Supplementary material for: Secondary Prevention of AFAIS: Deploying Traditional Regression, Machine Learning, and Deep Learning Models to Validate and Update CHA2DS2-VASc for 90-Day Recurrence
Source: J Clin Med. 2025 Oct 16;14(20):7327. doi: 10.3390/jcm14207327 (PMC12565214; doi:10.3390/jcm14207327)
Supplement: Supplementary file 1 [file jcm-14-07327-s001.zip › jcm-3802014-supplementary file S3 (appendix)-1.pdf]

Appendix

S1. Eligibility criteria (comparable items grouped across contributing trials)

- 18 or more years; 18 to 90 years; 18 to 80 years
- AIS of hemispheric localisation (excluding brainstem and cerebellum) of suspected thromboembolic origin; Moderate to severe ischaemic hemispheric stroke
- No pre-existing functional handicap; Previously independent
- Conscious; < 2 points on NIHSS for level of consciousness
- Clinical diagnosis of acute stroke within 6 h before entry into study; Clinical diagnosis of acute stroke within 12 h of treatment
- ≥ 6 on NIHSS at baseline; 8 to 23 on NIHSS at baseline
- ≥ 2 points on NIHSS for limb weakness; ≥ 3 points on NIHSS for sum of arm, leg, and hand strength items (5, 6, and 12); ≥ 2 points on NIHSS for motor deficit (for either one arm or leg); Limb weakness (drift within 10 s for the arm or 5 s for the leg; if mild, both should be affected)
- Symptoms of acute stroke; Higher cortical dysfunction plus homonymous visual field defect; At least one of: visual field deficit, neglect, or aphasia; Stable moderate to severe hemispheric stroke syndrome (defined as moderate to high-grade hemiparesis, sensory disturbance, dysarthria or nonfluent aphasia, and occasionally hemianopia)
- Treated within 4.5 h of symptom onset; Treated within 6 h of symptom onset
- No or only minor early infarct signs on the initial CT scan
- Adequate contraception for women of child-bearing potential
- Provision of informed consent according to local guidelines
- Patient could be followed up for the 90-day study period

S2. Recruitment periods of contributing trials

S2.1. AF dataset

**Accrual**		**Frequency (*n*)**	**Percentage (%)**
**Start**	**End**		
Late 1992	Early 1994	126	4.5
Oct 1996	Jan 1998	193	7.0
Mar 1998	May 1999	428	15.5
Apr 1998	Oct 1999	353	12.8
Sep 2002	Sep 2004	204	7.4
May 2003	Nov 2004	400	14.5
May 2003	Jun 2006	871	31.5
Not recorded		188	6.8
Total		2763	100.0

S2.2. Non-AF dataset

**Accrual**		**Frequency (*n*)**	**Percentage (%)**
**Start**	**End**		
Late 1992	Early 1994	575	7.4
Oct 1996	Jan 1998	621	8.0
Mar 1998	May 1999	1018	13.0
Apr 1998	Oct 1999	1007	12.9
Sep 2002	Sep 2004	503	6.4
May 2003	Nov 2004	1305	16.7
May 2003	Jun 2006	2370	30.3
Not recorded		410	5.3
Total		7809	100.0

S3. Setting of contributing trials

S3.1. AF dataset

**Setting**	**Frequency (*n*)**	**Percentage (%)**
Acute centre(s)	1185	42.9
Hospital	1578	57.1
Total	2763	100.0

S3.2. Non-AF dataset

**Setting**	**Frequency (*n*)**	**Percentage (%)**
Acute centre(s)	3355	43.0
Hospital	4454	57.0
Total	2763	100.0

S4. Location of centres participating in contributing trials

S4.1. AF dataset

**Location of centres**	**Frequency (*n*)**	**Percentage (%)**
Argentina, Austria, Australia, Belgium, Bulgaria, Brazil, Canada, Chile, China, Czech Republic, France, Germany, Greece, Hong Kong, Hungary, Israel, Korea, Mexico, Philippines, Poland, Portugal, Russia, Singapore, Slovakia,	871	31.5

South Africa, Spain, Switzerland		
Australia, Austria, Belgium, Denmark, Finland, France, Germany, Italy, Netherlands, New Zealand, Norway, Portugal, Spain, Sweden, Switzerland, UK	193	7.0
Australia, Belgium, Bulgaria, Czech Republic, Denmark, Finland, France, Germany, Hong Kong, Hungary, Italy, Malaysia, Netherlands, New Zealand, Norway, Poland, Portugal, Singapore, Slovakia, South Africa, South Korea, Spain, Sweden, UK	400	14.5
Austria, Australia, Belgium, Denmark, Finland, France, Germany, Greece, Hong Kong, Iceland, Israel, Italy, Netherlands, Norway, New Zealand, Portugal, South Africa, Singapore, Spain, Sweden, UK	428	15.5
Austria, Australia, Belgium, Canada, France, Finland, Germany, Hungary, Israel, Italy, Spain, Sweden, Netherlands, UK, US	204	7.4
Austria, Belgium, Denmark, Finland, France, Germany, Italy, Netherlands, Norway, Portugal, Spain, Sweden, Switzerland, UK	126	4.6
Canada, US	541	19.6
Total	2763	100.1

S4.2. Non-AF dataset

**Location of centres**	**Frequency (*n*)**	**Percentage (%)**

Argentina, Austria, Australia, Belgium, Bulgaria, Brazil, Canada, Chile, China, Czech Republic, France, Germany, Greece, Hong Kong, Hungary, Israel, Korea, Mexico, Philippines, Poland, Portugal, Russia, Singapore, Slovakia, South Africa, Spain, Switzerland	2370	30.3
Australia, Austria, Belgium, Denmark, Finland, France, Germany, Italy, Netherlands, New Zealand, Norway, Portugal, Spain, Sweden, Switzerland, UK	621	8.0
Australia, Belgium, Bulgaria, Czech Republic, Denmark, Finland, France, Germany, Hong Kong, Hungary, Italy, Malaysia, Netherlands, New Zealand, Norway, Poland, Portugal, Singapore, Slovakia, South Africa, South Korea, Spain, Sweden, UK	1305	16.7
Austria, Australia, Belgium, Denmark, Finland, France, Germany, Greece, Hong Kong, Iceland, Israel, Italy, Netherlands, Norway, New Zealand, Portugal, South Africa, Singapore, Spain, Sweden, UK	1018	13.0
Austria, Australia, Belgium, Canada, France, Finland, Germany, Hungary, Israel, Italy, Spain, Sweden, Netherlands, UK, US	503	6.4
Austria, Belgium, Denmark, Finland, France, Germany, Italy, Netherlands, Norway, Portugal, Spain, Sweden, Switzerland, UK	575	7.4
Canada, US	1417	18.1
Total	7809	99.9

S5. Sample size calculations for LR

S5.1. Sample size for estimation of prevalence of 90-day recurrence in target population with high precision

For a binary outcome, an approximate 95% confidence interval for the overall outcome proportion (**ϕ**) is,

$$\widehat{\phi} \pm 1.96\sqrt{\frac{\widehat{\phi}(1-\widehat{\phi})}{n}},$$

and so the absolute margin of error (δ) is $1.96\sqrt{\frac{\widehat{\phi}(1-\widehat{\phi})}{n}}$.

Thus, to aim for precise estimation of the prevalence of 90-day recurrence in the target population, based on the anticipated outcome proportion ($\widehat{\phi}$) and the desired margin of error, the required sample size is calculated as:

$$n = \left(\frac{1.96}{\delta}\right)^2 (\widehat{\phi})(1 - \widehat{\phi}).$$

Per Riley et al.'s recommendation [84], we aimed for a margin of error of ≤ 0.05. Then assuming an anticipated 90-day recurrence rate in the study population of 0.25,

$$n = \left(\frac{1.96}{0.05}\right)^2 (0.25)(1 - 0.25) = 288.12,$$

that is, at least 289 participants are needed to target an estimation error of at most 0.05 around the true value of 0.25.

S5.2. Sample size required to ensure a small mean absolute error in predicted probabilities when applied in other targeted individuals

Smeden et al. [84] derived the following formula;

$$ln(MAPE) = -0.508 - 0.544\,ln(n) + 0.259\,ln(\phi) + 0.504\,ln(P),$$

where n is the sample size of the development dataset, ϕ is the prevalence of 90-day recurrence in the target population, and P is the number of predictor variables. $MAPE$ denotes the mean absolute prediction error (i.e., the average error in the model's estimated 90-day recurrence probability one would allow for in the intended setting for application of the CPR).

Rearranging this equation and, per recommendation [84], choosing a target value of 0.05 for $MAPE$, we require:

$$n = exp(\frac{-0.508+0.259\, ln(\phi)+0.504\, ln(P)-ln(MAPE)}{0.544})$$

$$= exp(\frac{-0.508+0.259\, ln(0.25)+0.504\, ln(8)-ln(0.05)}{0.544}) = 343.62,$$

that is, at least 344 participants.

S5.3. Sample size required to target a small magnitude of required shrinkage of predictor effects

Riley et al. have shown that for binary outcomes, the sample size needed to achieve an expected uniform shrinkage factor S can be expressed as [84]:

$$n = \frac{P}{(S-1)\, ln(1-\frac{R^2_{CS}}{S})}.$$

They suggest targeting a shrinkage of ≤ 10% such that $S \geq 0.9$. Hence, to develop a logistic regression model based on our 8 predictor variables with an anticipated R^2_{CS} of at least 0.10, then to target an expected shrinkage of 0.9 we need a sample size of:

$$n = \frac{8}{(0.9-1)\, ln(1-\frac{0.1}{0.9})} = 679.21,$$

that is, <u>at least 680 participants</u>.

S5.4. Sample size required to target a small optimism in model fit

The aim here is to calculate the sample size required to ensure a small expected optimism in the apparent R^2 (i.e., $\frac{R^2_{CS}}{max(R^2_{CS})}$). This first requires the calculation of the shrinkage factor that corresponds to an expected optimism of δ in $R^2_{Nagelkerke}$. The solution provided by Riley et al. [84] is:

$$S = \frac{R^2_{CS}}{R^2_{CS} + \delta\, max(R^2_{CS})}.$$

Per recommendation, we employ a small value for δ, namely ≤ 0.05 [84]. The obtained value of S can then be placed into the aforementioned equation:

$$n = \frac{P}{(S-1)\, ln(1 - \frac{R^2_{CS}}{S})}.$$

The value of $max(R^2_{CS})$ is computed as follows:

$$max(R^2_{CS}) = 1 - exp(\frac{2\, ln(L_{null})}{n}),$$

where L_{null} is defined by

$$L_{null} = E\, ln(\frac{E}{n}) + (n - E)\, ln(1 - \frac{E}{n}),$$

E being the total number of patients with 90-day recurrence present in our dataset and n our arbitrarily chosen sample size. Hence, we have

$$ln(L_{null}) = (0.05 \times 2763)\, ln(\frac{0.06\times 2763}{2763}) + (2763 - (0.05 \times 2763))ln(1 - \frac{0.05 \times 2763}{2763})$$

$$= -548.50, \text{ and}$$

$$max(R^2_{CS}) = 1 - exp(\frac{2 \times (-1553.73)}{2763}) = 0.675.$$

Substituting these values, we obtain the following value for S:

$$S = \frac{0.10}{0.10 + (0.05\times 0.328)} = 0.859.$$

This leads to a required sample size of:

$$n = \frac{8}{(0.859-1)\, ln(1-\frac{0.10}{0.859})} = 458.42$$

that is, at least 459 participants.

S6. Worked example of CPR development, described in section 12.4.7

Step 1. SHAP-to-point conversion

Compute the mean SHAP value of each of the eight features. Rank the features from most to least important, sorting them into three tiers: (1) the lower tier grouping features with mean SHAP values below the 25th percentile, (2) the middle tier grouping features with mean SHAP values between the 25th and 75th percentiles, and (3) the upper tier grouping features with mean SHAP values above the 75th percentile. Features belonging to the lower, middle, and upper tier are assigned 1, 2, and 3 points, respectively. This process is tabulated in an example below.

**Feature**	**Mean SHAP value**	**Percentile rank**	**Tier**	**Points assigned**
Age ≥ 75	0.082	90th	Upper	3
Age 65–74	0.047	75th	Upper	3
CHF	0.043	60th	Middle	2
Hypertension	0.036	55th	Middle	2

Diabetes mellitus	0.021	35th	Middle	2
Stroke history	0.018	30th	Middle	2
Vascular disease	0.010	15th	Lower	1
Female sex	–0.004	<0	Excluded	0

Step 2. Interaction-augmentation

Compute the mean SHAP interaction value for each unique feature pair among the eight features (28 pairs). Rank pairs from most to least important and retain those at or above the 90th percentile. Each retained pair contributes a +1 interaction bonus when both features are present. This process is tabulated in an example below.

**Feature pair**	**Mean interaction SHAP value**	**Percentile rank**	**Included in CPR**	**Bonus points**
Age ≥ 75, CHF	0.025	95th	Yes	+1
Hypertension, DM	0.020	92nd	Yes	+1

CHF, DM	0.018	91st	Yes	+1
All other pairs	<0.015	<90th	No	0

Step 3. Compute patient score

Patient A has: age $\geq$ 75, CHF, hypertension, DM.

Main effects: 3 (Age) + 2 (CHF) + 2 (HTN) + 2 (DM) = 9.

Interaction bonuses: Age, CHF (+1), HTN, DM (+1), CHF, DM (+1) = +3.

Hence, the total CPR score for patient A = 12.
